# ProGenExpress: Visualization of quantitative data on prokaryotic genomes

**DOI:** 10.1186/1471-2105-6-98

**Published:** 2005-04-13

**Authors:** Michael Watson

**Affiliations:** 1 Institute for Animal Health, Compton laboratory, High street, Compton, Newbury, RG20 7NN, UK

## Abstract

**Background:**

The integration of genomic information with quantitative experimental data is a key component of systems biology. An increasing number of microbial genomes are being sequenced, leading to an increasing amount of data from post-genomics technologies. The genomes of prokaryotes contain many structures of interest, such as operons, pathogenicity islands and prophage sequences, whose behaviour is of interest during infection and disease. There is a need for simple and novel tools to display and analyse data from these integrated datasets, and we have developed ProGenExpress as a tool for visualising arbitrarily complex numerical data in the context of prokaryotic genomes.

**Results:**

Here we describe ProGenExpress, an R package that allows researchers to easily and quickly visualize quantitative measurements, such as those produced by microarray experiments, in the context of the genome organization of sequenced prokaryotes. Data from microarrays, proteomics or other whole-genome technologies can be accurately displayed on the genome. ProGenExpress can also search for novel regions of interest that consist of groups of adjacent genes that show similar patterns across the experimental data set. We demonstrate ProGenExpress with microarray data from a time-course experiment involving *Salmonella typhimurium*.

**Conclusion:**

ProGenExpress can be used to visualize quantitative data from complex experiments in the context of the genome of sequenced prokaryotes, and to find novel regions of interest.

## Background

The genomes of prokaryotic organisms contain many structures that may be involved in pathogenicity, including a variety of operons, pathogenicity islands and prophage sequences. Operons are sets of adjacent genes in bacteria that form a single transcriptional unit, and many, such as those coding for flagella [1] or fimbriae [2], have been implicated in pathogenicity. Pathogenicity islands are distinct regions of the genome that confer virulence upon the host, and are found in many pathogens of humans, animals and plants, and at least ten pathogenicity islands have been identified in *Salmonella *alone [3]. Prophage sequences represent the chromosomes of bacteriophage integrated as part of the genome of the bacterial host, and have also been implicated in pathogenicity in several species [4].

In order to study the behaviour of these elements, it is essential to integrate information about the genome structure of an organism with quantitative measurements produced by post-genomic technologies, such as those from microarray or proteomics experiments. This integrative biology approach is a key feature of systems biology. Studying the behaviour of these genomic elements, and other groups of adjacent genes, during infection and disease may reveal important information about the molecular mechanisms underlying pathogenicity.

Several microbial genome viewers have been developed which allow quantitative data to be displayed on the genome. The **Microbial Genomes Viewer **[5] offers a good online solution, however users must install a browser plug-in and may not be comfortable transmitting data over the internet. **GenoMap **[6] can be used to create plots of microarray data on microbial genomes, and is available as Tcl/TK source code. **Genome2D **[7] also offers good visualisation of quantitative data on microbial genomes, but is limited to the Windows operating system. Finally, **GenomeViz **[8] has recently been released, which offers much functionality, including visualisation of quantitative data, genome alignments and GC content. However this software is currently limited to unix-based systems. **All **of the above solutions are limited in two respects. Firstly, the quantitative values are represented as a colour-scale, which reduces the accuracy of the data and which may present problems in comparing one colour to the next. Secondly, the above tools can only display a single value for each gene, which precludes the visualisation of more complex data, such as a time-course experiment.

## Implementation

ProGenExpress is released as a package for R. R is a freely available, open-source statistical package [9] that is widely used in the biological community. R has very powerful statistical and graphical capabilities, and many add-on packages are freely available. The bioconductor project [10,11] provides a huge number of add-on packages for R, covering a wide range of biological data analysis applications, and the implementation of ProGenExpress in R provides seamless integration with many of these packages. ProGenExpress is written in the native R language and has been fully tested on both windows and linux. R is available for windows, linux, unix and MacOS (including MacOS X).

## Results and discussion

ProGenExpress has been written to allow researchers to quickly and simply visualize the behaviour of bacterial genomic regions of any size during experiments using whole genome technologies, such as microarray or proteomics experiments. For information relating to the genome organisation of prokaryotes, ProGenExpress includes functions for downloading and reading both NCBI .ptt files, which describe the location of protein coding genes in bacteria in a tabular format, and include links to the COGs database [12], and whole genome RefSeq entries [13]. For the quantitative experimental data, ProGenExpress can use the objects created by many of the packages from the bioconductor project [10,11], or data imported into R from text files, SQL databases and Excel.

There are currently 225 completed prokaryotic genomes in RefSeq [15] that ProGenExpress can read, and though the utility of ProGenExpress is demonstrated here using microarray data, any kind of numerical data that can be linked to the genes of prokaryotic organisms can be displayed using ProGenExpress. Where measures of the statistical significance of the data points for each gene are available, these can be passed to the plotting functions of ProGenExpress, with the result that those genes that are not significant will be plotted in white and those that are significant will be plotted in their normal plotting colour.

The genome is represented as two barplots, one for each strand. Each gene has a number of bars equal to the number of experimental data sets included, allowing time-course or complex strain/treatment experiments to be plotted. Distance between the bars for each gene is representative of intergenic distance. Slices of the genome can be selected either by base range, gene synonym or gene name. Both horizontal and vertical plots are possible, and bars can be coloured either by numerical value or by COGs [12] functional category.

The software is demonstrated here using microarray data from Eriksson *et al *[14]. This data set consists of gene expression measurements from intracellular *Salmonella typhimurium *at 4, 8 and 12 hours post murine macrophage infection. Gene expression values were calculated as the relative expression level of test RNA to that of RNA from bacteria grown *in vitro*, and the data has been centred and normalised according to Eriksson *et al *[14]. Data from Erikson *et al *is available as a spreadsheet [14]. This spreadsheet was pre-processed to contain only columns for gene synonym, gene name and relative expression level of test RNA to control RNA on a log 2 scale for each of the three time points. The spreadsheet was saved as tab-delimited text and read in to R using the read.table() function. The S typhimurium genome and plasmid sequences were read in to R using the read.ptt() function, with RefSeq files NC_003197.ptt and NC_003277.ptt respectively. The microarray data was linked to the gene location data using the linkem.avg() function. Images of the microarray data on both the entire genome and the plasmid were then generated using the plotrange() and plotrange.vertical() functions in conjunction with jpeg(), an internal R function. The results were viewed in Internet Explorer. Finally, the find.region() function was used to find regions of interest as described below.

**Figure 1:**
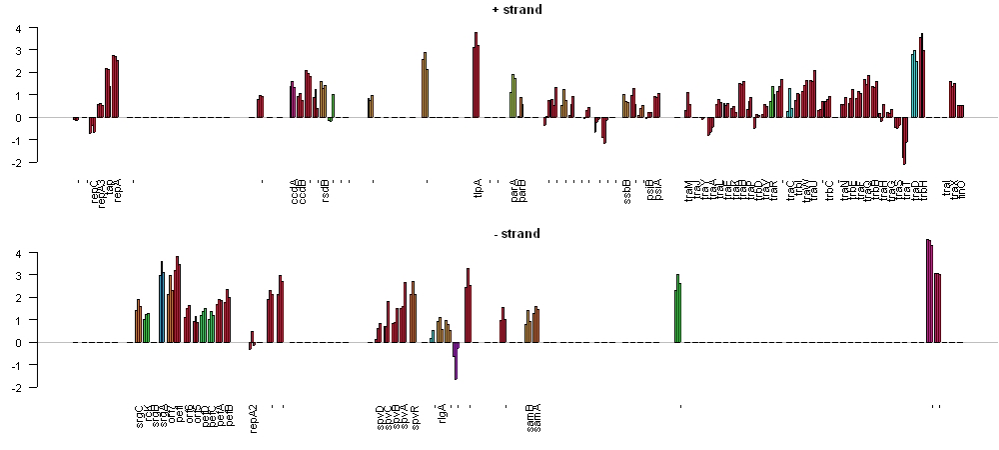
**Gene expression measurements for *Salmonella typhimurium *plasmid pSLT**. Gene expression measurements for the pSLT plasmid of *Salmonella typhimurium*. The genome is represented as two barplots, one for each strand. Each gene has three bars representing expression at 4 h, 8 h and 12 h post macrophage infection. Gene expression measurements (y-axis) are ratios of test RNA to control RNA on the log 2 scale. Bars are colour-coded according to the COGs [12] functional category. Distance between genes is relative and representative of intergenic distance. This image clearly shows that the majority of genes on the plasmid are up-regulated during macrophage infection.

**Figure 2:**
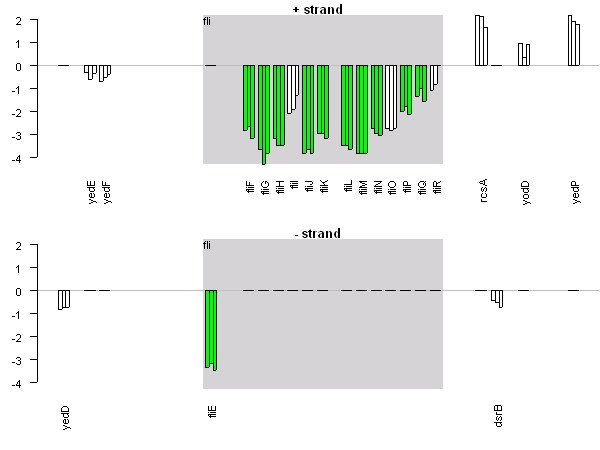
**Gene expression measurements for flagella biosynthesis**. Gene expression measurements for the fli operon from *Salmonella typhimurium*. The genome is represented as two barplots, one for each strand. Each gene has three bars representing expression at 4 h, 8 h and 12 h post macrophage infection. Gene expression measurements (y-axis) are ratios of test RNA to control RNA on the log 2 scale. Distance between genes is relative and representative of intergenic distance. The fli operon is involved in flagella biosynthesis and this image clearly shows that the entire operon is strongly down-regulated during macrophage infection. Significantly differentially expressed genes are coloured red or green, whereas non-significant genes are coloured white. All but three of the 14 genes in the operon are significantly differentially expressed.

**Figure 3:**
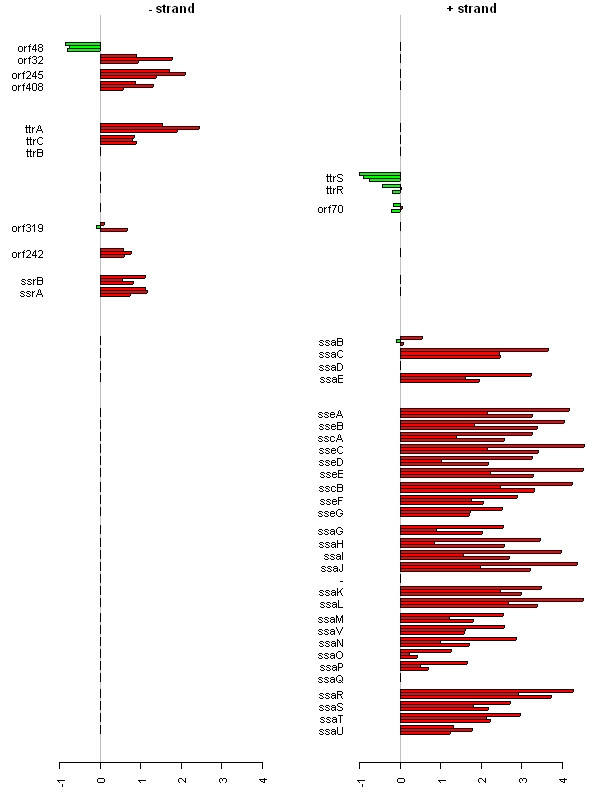
**Gene expression measurements for Salmonella pathogenicity island II**. Gene expression measurements for Salmonella Pathogenicity Island II (SPI-II) from *Salmonella typhimurium*. The genome is represented as two barplots, one for each strand. Each gene has three bars representing expression at 4 h, 8 h and 12 h post macrophage infection. Gene expression measurements (y-axis) are ratios of test RNA to control RNA on the log 2 scale. Distance between genes is relative and representative of intergenic distance. This island has been linked to pathogenicity, and encodes a type III secretion system. It is required for systemic infection and intracellular pathogenesis by facilitating replication of intracellular bacteria within membrane-bound Salmonella-containing vacuoles [3]. Here we can clearly see that the majority of genes in the island are strongly up-regulated during macrophage infection.

Figure 1 shows the expression of all genes on *Salmonella typhimurium *LT2 plasmid pSLT, coloured by COGs functional category. The majority of the genes on this plasmid are up-regulated at all three time-points, implying a role for this plasmid during macrophage infection. Figure 2 displays a smaller region of the genome containing the fli operon, with all genes in the operon displaying similar expression profiles. Erikksson *et al *[14] found 919 genes to be significantly differentially expressed, and that measure of statistical significance has been incorporated into Figure 2. Significant genes are coloured normally, whereas those that are not significant are white. All but three of the 14 genes in the operon are shown to be significantly differentially expressed, suggesting that the whole operon is differentially expressed and that perhaps the measure of statistical significance used is too stringent. Finally, Figure 3 is a vertical plot of *Salmonella *pathogenicity island II (SPI-II), showing that most genes on this island are up-regulated at all three time-points. This island encodes a type III secretion system, and has been shown to be required for systemic infection by facilitating replication of intracellular bacteria within membrane-bound Salmonella-containing vacuoles [3].

**Figure 4:**
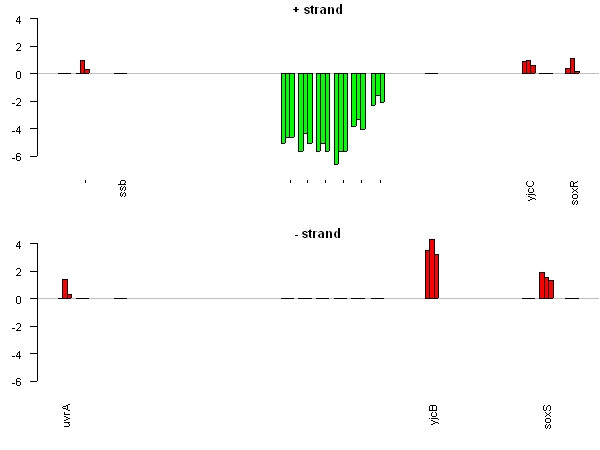
**A putative operon**. Gene expression measurements from a genomic region of *Salmonella typhimurium*. The genome is represented as two barplots, one for each strand. Each gene has three bars representing expression at 4 h, 8 h and 12 h post macrophage infection. Gene expression measurements (y-axis) are ratios of test RNA to control RNA on the log 2 scale. Distance between genes is relative and representative of intergenic distance. The image shows six genes that have been identified by ProGenExpress as potentially interesting. The genes are very close together on the genome and display similar expression patterns, and therefore could represent an as yet unannotated operon. The genes have synonyms STM4257 – STM4262 and currently have no confirmed function.

ProGenExpress can also search for operons and other regions of interest by looking for clusters of genes that are close together and which display similar patterns in the experimental data. Using this facility, we identified over 200 potential regions of interest in *Salmonella typhimurium *consisting of four genes or more, including several known operons and potential unannotated operons. Figure 4 shows a region of the genome containing a group of six genes that has been found using ProGenExpress. The genes have no assigned gene name, have either an unknown or putative/predicted function, are close together on the genome and have similar expression profiles across the three time-points. We believe these genes may represent an unannotated operon.

ProGenExpress has several advantages over existing software. The package seamlessly integrates with the bioconductor project and the many packages available in R for microarray analysis, including limma, marray and affy, and is available for both Windows and Linux, amongst others. Both horizontal and vertical plots are possible, and an unlimited number of data points for each gene can be plotted, allowing for the visualization and analysis of complex time course or strain/treatment experiments. Furthermore, the bar-plots display numerical data accurately, and do not rely on a colour-scale to depict values. Finally, the ability to search integrated genomic and post-genomic data sets for clusters of genes which behave similarly represents an opportunity for the discovery of novel genomic elements involved in pathogenicity.

## Conclusion

We describe ProGenExpress, an open-source R package which allows researchers to quickly and easily visualise quantitative data from arbitrarily complex experiments in the context of the genome of sequenced prokaryotes. ProGenExpress can also be used to search for genomic regions which may represent coherent functional units. We show how ProGenExpress can be used to visualise microarray data from a time-course experiment on the genome of *Salmonella typhimurium*, and to find unannotated genomic regions that may be involved in pathogenicity. Future plans for the software include the ability to read data from ensembl databases, and the development of visualisation tools for eukaryotic genomes. Software updates and new releases will be available from the project home page.

## Availability and requirements

• **Project Name: **ProGenExpress

• **Project Home Page**: 

• **Operating Systems: **Windows, Linux, Unix

• **Programming Language: **R

• **Other Requirements: **R version 2.0 or above

• **License: **GNU GPL

## Authors' contributions

MW developed and tested the software in full.

## List of abbreviations

COG: Cluster of Orthologous Groups

SPI-II: *Salmonella *pathogenicity island II
